# Supplementary material for: Synthesis of Monolayer MoSe2 with Controlled Nucleation via Reverse-Flow Chemical Vapor Deposition
Source: Nanomaterials (Basel). 2019 Dec 31;10(1):75. doi: 10.3390/nano10010075 (PMC7023349; doi:10.3390/nano10010075)
Supplement: Supplementary file 1 [file nanomaterials-10-00075-s001.pdf]

Supplementary Information

Synthesis of Monolayer $MoSe_2$ With Controlled Nucleation via Reverse-Flow Chemical Vapor Deposition

Siyuan Wang [1,2,†], Guang Wang [2,†,*], Xi Yang [3], Hang Yang [2], Mengjian Zhu [3], Sen Zhang [2], Gang Peng [2] and Zheng Li [1,*]

[1] School of Material Science and Engineering, Xiangtan University, Xiangtan 411105, China
[2] College of Liberal Arts and Sciences, National University of Defense Technology, Changsha 410073, China
[3] College of Advanced Interdisciplinary Studies, National University of Defense Technology, Changsha 410073, China
* Correspondence: wangguang@nudt.edu.cn (G.W.); lizheng@xtu.edu.cn (Z.L.)
† Siyuan Wang and Guang Wang contributed equally to this work.

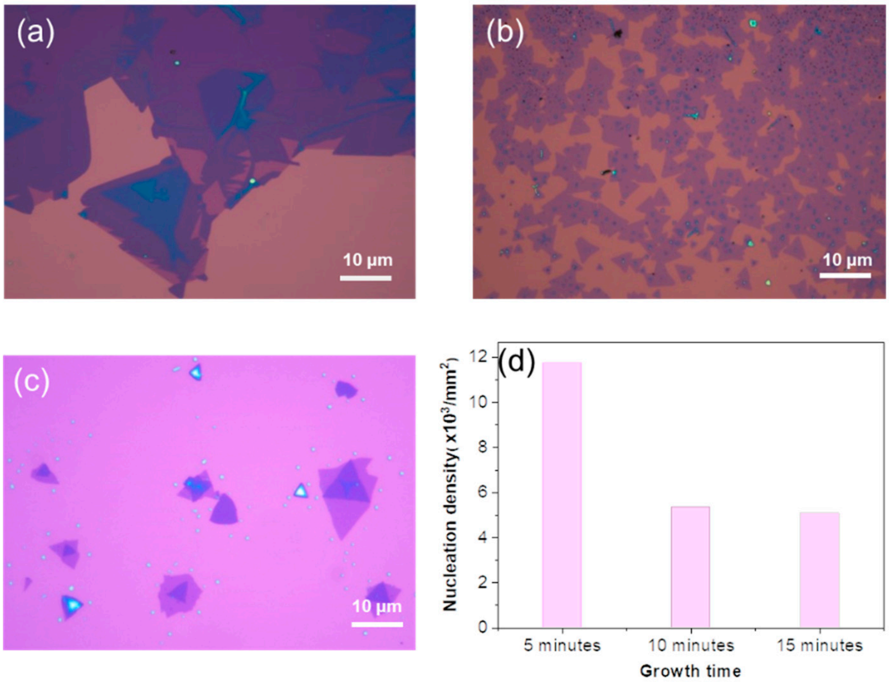

Figure S1 Optical images of $MoSe_2$ flakes (a) with poor uniformity and (b) excessive nucleation with forward-flow. (c) uniform morphology without optimized reverse-flow. (d) Variation of nucleation density with growth time.

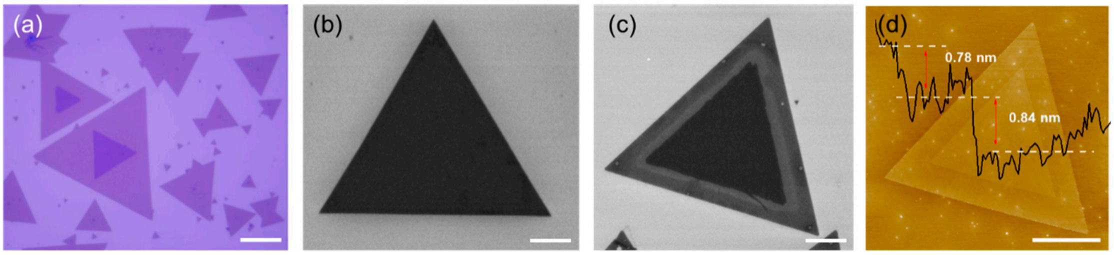

Figure S2 (a) Optical images of $MoSe_2$ flakes. SEM images of (b) monolayer and (c) bilayer $MoSe_2$ flakes. (d) AFM image shows height profile of the bilayer $MoSe_2$ flake.

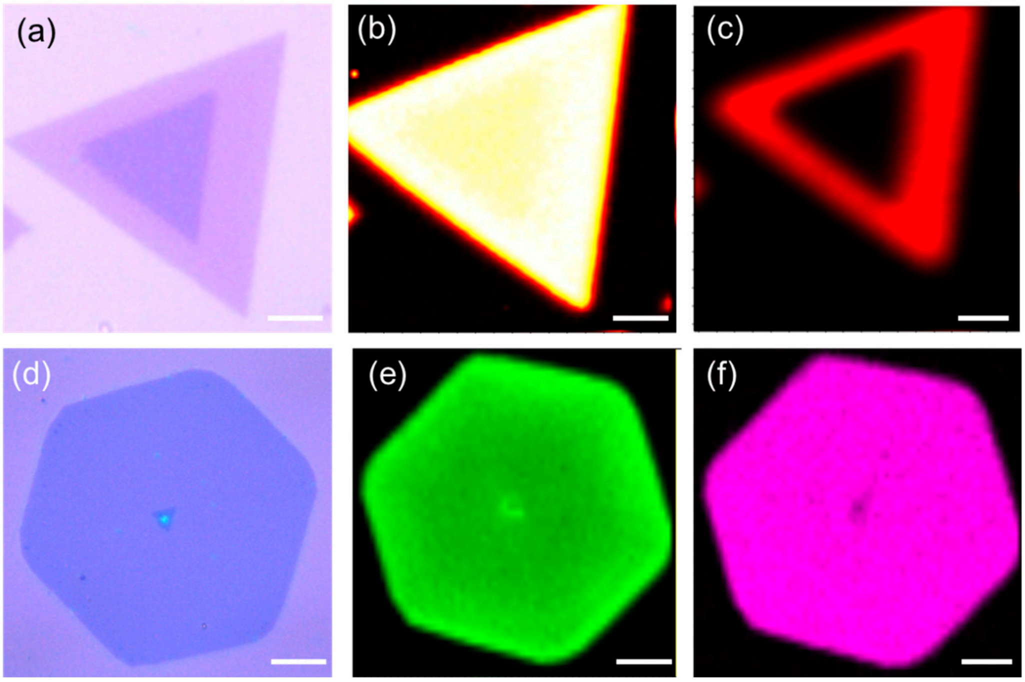

Figure S3 (a) The optical image, (b) Raman (A_{1g} mode, 240 cm^{-1}) and (c) PL (807 nm) peak intensity map of a triangular bilayer $MoSe_2$ flake, respectively. (d) The optical image, Raman peak intensity map of (e) A_{1g} mode (240 cm^{-1}) and (f) E_{2g}^{1} mode (287 cm^{-1}) of a hexagonal $MoSe_2$ flake, respectively. The scale bar is 10 μm.

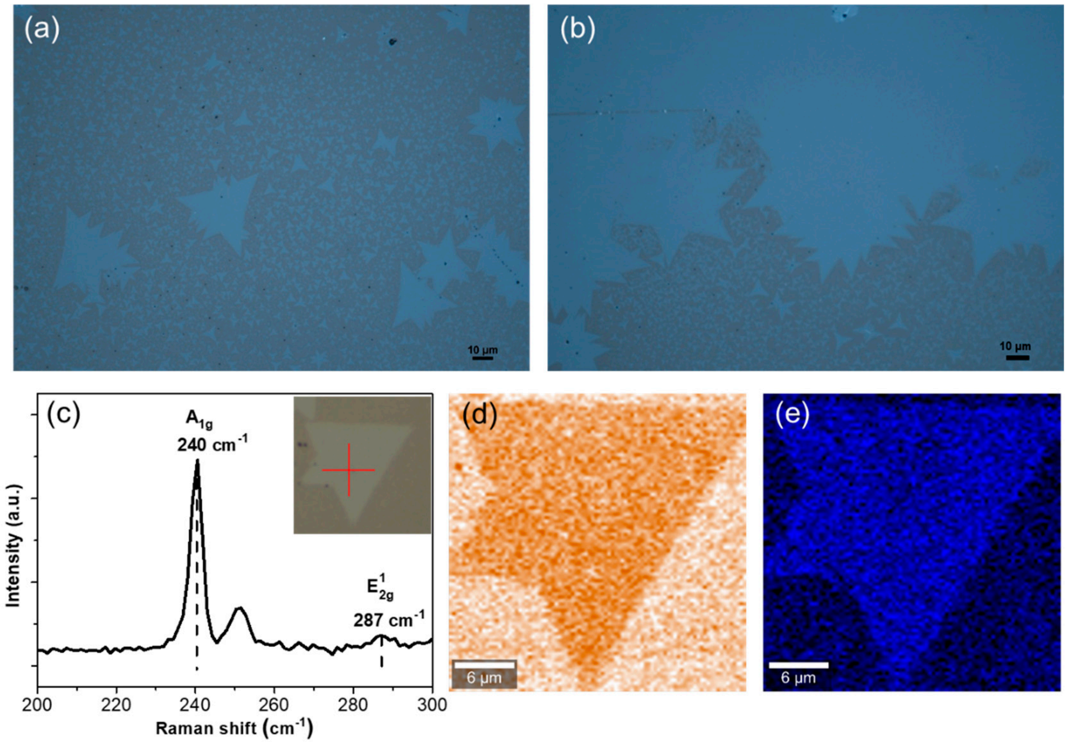

Figure S4 The optical images of monolayer (a) $MoSe_2$ flakes and (b) $MoSe_2$ films grown on sapphire (0001) substrate. (c) Raman spectrum of monolayer $MoSe_2$ grown on sapphire substrate. Raman peak intensity map of (d) A_{1g} mode (240 cm^{-1}) and (e) E_{2g}^{1} mode (287 cm^{-1}) of monolayer $MoSe_2$ flake, respectively.
